# Fifty Years of *One Flew Over the Cuckoo's Nest*: A Qualitative Exploration of Mental Health Staff's Perspectives

**DOI:** 10.1111/inm.70181

**Published:** 2025-11-25

**Authors:** John Goodwin, Katerina Drakos

**Affiliations:** ^1^ Catherine McAuley School of Nursing and Midwifery University College Cork Cork Ireland; ^2^ Child and Adolescent Mental Health Center University Hospital–Mental Health Services CPH Copenhagen Denmark; ^3^ Copenhagen Center for Social Data Science (SODAS) University of Copenhagen Copenhagen Denmark

**Keywords:** film, media, mental health, psychiatry, stigma

## Abstract

In 2025, the film *One Flew Over the Cuckoo's Nest* turned 50 years old. Although the film has been subject to analysis over the years, its role in shaping the perceptions of professionals working in mental health has not yet been fully explored. The aim of this paper is to explore mental health staff's perspectives on*One Flew Over the Cuckoo's Nest* on its 50th anniversary. Semi‐structured interviews were conducted with 15 participants. The study was guided by a qualitative descriptive approach. Reflexive thematic analysis was used to analyse data. Five Themes were identified: Personal Reflections on First Viewing *One Flew Over the Cuckoo's Nest*, Consolidating Stereotypes and Reinforcing Stigma, The Shadow of Nurse Ratched, We Have Come a Long Way but We Still Have a Long Way to Go and The Legacy of *One Flew Over the Cuckoo's Nest* Today. Although the film's direct influence is waning, it still has some hold over the public's negative views of mental health care. Media campaigns, similar to those used to dispel myths about mental health challenges, should be used to promote better images of mental health staff and care environments, reducing stigma. While mental health care has come a long way since the release of *One Flew Over the Cuckoo's Nest*, further improvements are warranted, with an emphasis on reducing power dynamics and more focused mental health education.

## 1 Introduction

The year 2025 marked the 50‐year anniversary of the film *One Flew Over the Cuckoo's Nest* (Forman 1975), based on the book by Ken Kesey, published in 1962. The book itself is regarded as a foundational work of American literature and when released left a long‐lasting impression on its readers (Stip et al. 2023). There have been several scholarly works dedicated to analysing the film and its influence on the public, with many noting how it has fostered stigma towards mental illness and mental health care (Cabrera et al. 2021; Hamilton 2024). However, there is a limited understanding as to how *One Flew Over the Cuckoo's Nest* has shaped mental health staff's perceptions. Using a qualitative descriptive approach with international participants from different professional backgrounds, this paper explores health staff's perspectives on *One Flew Over the Cuckoo's Nest* on its 50th anniversary.

## 2 Background

*One Flew Over the Cuckoo's Nest* tells the story of Randle P. ‘Mac’ McMurphy (played by Jack Nicholson), who is a patient at a mental institution and his dramatic rebellion against the ‘Big Nurse’/Nurse Ratched (played by Louise Fletcher) (Domino 1983; Voyce 2025). It has been critically acclaimed and is widely regarded as one of the greatest films of all time, exploring complex topics of mental illness, institutional power relations and autonomy (Abdulkadhim Neamah Alrubaye 2024). In the 1970s, the theme of freedom against authority was timely as the first steps towards deinstitutionalisation were taking place across the world which meant that the defiant stance against the psychiatric system created a sense of heroism in McMurphy's character (Anderson 2003; Rondinone 2020)—this is important given the influence that media can have on the general public (Goodwin et al. 2023; Liu 2023).

Media representations can shape public views of complex topics, with the filmmaker/artist invoking symbols, metaphors, dialogue and aesthetic moments to explore a difficult topic. Popular cinema as a medium also allows for reflection on opinions, attitudes and beliefs on these topics (Aventin et al. 2019; Liu 2023). According to Bateson's (1972) Framing Theory, the way information is framed within the media, including film, influences public opinion. Moreover, Cultivation Theory (Gerbner and Gross 1976) proposes that exposure to multimedia shapes how the public perceive reality, or that people's world views align with what is presented through mediums such as film. On the other hand, Cavell (1979) viewed audiences as taking a more active stance when engaging with film, whereby reflection is encouraged, prompting questions and scepticism about the reality presented within filma. Bordwell and Thompson (2021) concur with regard to the reflective nature of film, emphasising that cinema is not merely a passive recording of reality and acknowledging the intentionality of the filmmakers when producing films. These theoretical approaches underscore the various ways in which film as a medium represents reality and how audiences are influenced by the medium's message and are encouraged to reflect on the messages within.

In relation to the empirical literature, recent evidence indicates that films can influence people's perceptions of a range of issues, including career pathways (Geena Davis Institute on Gender in Media 2018; Kool et al. 2022), smoking behaviours (Li et al. 2024) and climate change (Schneider‐Mayerson et al. 2025). Film can also have an influence on how the public perceive mental health, with perceptions of this area often negative (Anderson 2003; Hecht et al. 2022; Parrott 2023). Many authors have commented that *One Flew Over the Cuckoo's* *Nest*, in particular, has propagated myths and stereotypes about mental health, leading to spreading of misinformation about mental health care environments, staff and treatments (Anderson 2003; Bock et al. 2022; Cabrera et al. 2021).

One such detail of the film that has been widely explored is the public's negative perceptions of electroconvulsive therapy (ECT). In the film, McMurphy is subjected to ECT—without anaesthesia or muscle relaxants—as a consequence of not following the rules of the ward, as laid down by Nurse Ratched. Fifty years since its release, *One Flew Over the Cuckoo's Nest* remains the most frequently cited film depicting ECT (Cabrera et al. 2021; McFarquhar and Thompson 2008). Mental health professionals have been critical of the film's depiction of ECT, which was said to increase stigma towards the treatment and created attitudes that persist, despite evidence regarding the safety and efficacy of ECT (Cabrera et al. 2021; Hamilton 2024). In a qualitative study, when asked directly about ECT, members of the public and those with a history of depression cited the film as an explicit influence on their perceptions, and described the procedure as scary, traumatic or intense (Cabrera et al. 2021). Moreover, support declines among medical students after viewing clips of ECT from the film (McFarquhar and Thompson 2008). McDonald and Walter (2009) note that there are valid criticisms of ECT, but the film has convinced many members of the public that this is a one‐sided argument, and that any use of ECT is only ever coercive.

Although the impact of *One Flew Over the Cuckoo's Nest* on the public has been considered to have had a mostly negative effect towards mental illness and mental health care, some studies have shown the potential to educate young health professionals. The film can be used as a catalyst for deeper discussions on mental health practices and developments of facilities and care in the present day (Bock et al. 2022). Given the depiction of Nurse Ratched—a cold, strict, authoritarian figure—*One Flew Over the Cuckoo's Nest* has been used to explore mental health issues in nurse education (McCann and Huntley‐Moore 2016). The ‘Madness in the Movies’ module was implemented in a higher education institution in Ireland. Throughout the module, students compared and contrasted historical portrayals of mental health issues with the contemporary mental health landscape, attitudes and person‐centred practices within mental healthcare settings. Films, including *One Flew Over the Cuckoo's Nest* were used as a basis to discuss developments in mental health. Students felt that their understanding of mental health issues was enriched through their participation in the module, and they were made more aware of current policy initiatives in mental health care, especially considering the shift towards user‐led services, the recovery ethos and the importance of family involvement (McCann and Huntley‐Moore 2016).

Although both the book and the film have been analysed in depth by scholars, there is a gap in the research, in that its role in shaping the perceptions of professionals working in mental health has not yet been fully explored. Understanding these perceptions is important, given how media portrayals of mental health can influence stigma, treatment expectations (Goodwin et al. 2023, 2024), and staff's professional identities (Wu et al. 2024). The aim of this paper is to explore mental health staff's perspectives on the film *One Flew Over the Cuckoo's Nest* on its 50th anniversary.

## 3 Methods

### 3.1 Design

This study was guided by a qualitative descriptive approach. In such studies, the researcher is encouraged to adhere closely to participant data (Bradshaw et al. 2017). Taking such an approach facilitates the reporting of participants' perceptions of phenomena in their own words (Searby et al. 2022). This design was chosen as it allows for a nuanced overview of how people from diverse international contexts make sense of cultural material (such as film) (Kim et al. 2017; Neergaard et al. 2009; Sandelowski 2000). This was important to the current study, as *One Flew Over the Cuckoo's Nest* is a US production directed by a Czech‐American, the researchers are from Ireland and Portugal, and a diverse range of international participants was sought.

### 3.2 Data Collection

A purposeful sampling approach was adopted. Following ethical approval from the Social Research Ethics Committee, University College Cork, emails advertising the study were sent through the researchers' contact networks and advertised over social media. Interested parties contacted the researcher directly to arrange interviews.

Interviews were conducted online by an experienced mental health nurse academic (JG), via Microsoft Teams in January/February 2025; field notes were also recorded. Automatically generated transcripts were checked against the original audio for accuracy and then deleted. Interviews lasted 20–47 min. The interview guide is included as supplemental material.

### 3.3 Data Analysis

Braun and Clarke's (2021) reflexive thematic analysis was used to analyse data, facilitated by NVivo 14 (QSR International Pty Ltd. 2023). Transcripts were read numerous times, aiding immersion. Data were then coded by JG Next, themes were independently identified from codes by JG and KD These were then discussed, debated and refined before write‐up.

Lincoln and Guba's (1985) principles were followed to maintain rigour. The researchers had extensive research experience, with the lead researcher holding qualifications in mental health nursing and film studies, thus strengthening credibility (Jahn et al. 2025). Dependability and confirmability were enhanced by describing all steps of the research process in detail. In line with transferability, quotations from every participant interviewed are provided (Lincoln and Guba 1985; Vellakkal et al. 2017).

## 4 Results

Interviews were conducted with 15 participants; pseudonyms are used to protect anonymity. Most (*n* = 8) participants were female, resided in Ireland (*n* = 10), and came from nursing backgrounds (*n* = 14). A diverse range of areas practice were included: acute care, community care, education, forensics and the child and adolescent mental health services. Demographics are presented in full in Table 1.

**TABLE 1:** Demographics.

Characteristics	*n*
Gender	
Male	6
Female	8
Prefer to self‐describe (agender)	1
Age range	
18–25	1
31–35	6
36–40	2
41–45	3
46–50	2
51–55	1
Country of residence	
Ireland	10
New Zealand	2
Australia	2
Turkey	1
Profession	
Nursing	14
Clinical psychology	1
Area of practice	
Acute	3
Acute (children)	2
Community	2
Education	5
Forensics	3

Five themes were identified: Personal reflections on first viewing *One Flew Over the Cuckoo's Nest*, Consolidating Stereotypes and Reinforcing Stigma, The Shadow of Nurse Ratched, We Have Come a Long Way but We Still Have a Long Way to Go and The Legacy of *One Flew Over the Cuckoo's Nest* Today.

### 4.1 Theme 1. Personal Reflections on First Viewing *One Flew Over the Cuckoo's Nest*

Under this theme, participants spoke about when they first saw *One Flew Over the Cuckoo's Nest* and what struck them about it initially. Although some participants saw the film before the start of their healthcare studies, many were exposed to it during their college experience. This film was either screened during class‐time, or they sought it out after a reference was made to it in class:

I probably first saw it, I'd say three or four years ago, kind of in the middle of college studying to be a nurse (Gina)

Unanimously, participants considered it to be an excellent film, despite some of negative ways which in which mental illness and mental health care is framed. The film was considered to be very impactful, with certain images etched into participants' minds:

I think you don't need to watch it more than once or twice ‘cause it sticks in your mind’ (Fred)

Most participants had not been on clinical placement when they first saw the film. As such, the ‘reality’ the film represented about mental health care was accepted as authentic at the time. For some, this created a sense of fear about the clinical placements they would undertake when they were students:

I went to do my mental health placement: that's all I could think about. And I thought, ‘Oh my God, they're all going to be crazy. They're all going to be shouting and yelling at each other. They're all going to be trying to kill themselves and each other’, and I was terrified going into a mental health acute ward (Ivy)

### 4.2 Theme 2. Consolidating Stereotypes and Reinforcing Stigma

This theme reports on how the film has played a substantial role in contributing to mental health stigma in society. Participants commented that many members of the general public hold stigmatising beliefs about mental illness and mental health care, viewing service users through a stereotypical lens. *One Flew Over the Cuckoo's Nest* amplified and confirmed perceptions already held, intensifying stigma:

I think it probably reinforced stereotypes that people had and probably reinforced stigma (Anthony)

Images of service users projected by the film were of social outcasts, people who were potentially dangerous. Such individuals would be best segregated from regular society:

I think it probably again reaffirmed all the kind of prejudices that they're odd, weird kind of characters, that they're probably a little bit dodgy and best off avoided. And to some extent they need controlling (Dylan)

The sense of ‘danger’ linked with service users also extended to the hospital environment. Participants considered that the hospital in the film was portrayed more like a prison than a therapeutic environment. Rather than adopting a recovery‐orientated approach to care, a more punitive approach was depicted:

And the movie is very much put across like he's in, you know, it's a prison. And if you step out of line, you're punished and, like, punished very, very hard (Oliver)

Such punishments included the forced use of ECT and medication. Participants commented that the stark images of such treatment in the film have had an impact on society, with many of the public believing to this day that the approach is representative of the current mental health ethos:

People think that every time every type of mental illness will result in you ending up in a facility like *One Flew Over the Cuckoo's Nest* and being given ECT without your consent with no muscle relaxants and, you know, forced with medication (Hector)

I really think that people still think this is what mental health is still, like, that there's these big men ready to come in and flatten people and inject them when they misbehave (Janice)

Consequently, several participants commented that, over the years, the film has influenced people experiencing mental health challenges to avoid help‐seeking:

I don't want to go there because I'm not that. That's not me. I don't want to be with people like that. I don't want to be treated like that (Esme)

Because if you see that and you think that's what I'm going into, you're not going to go to place like that (Chris)

Despite consolidating stereotypes and reinforcing stigma, one participant highlighted one of the potential benefits of the film. *One Flew Over the Cuckoo's Nest* put mental health into the public consciousness, instigating conversations which has ultimately led to a more progressive approach today:

I think whilst it was shocking and whilst it was maybe not a true depiction of mental health, it did get mental health on the map a long time ago (Ivy)

### 4.3 Theme 3. The Shadow of Nurse Ratched

Under this theme the character of Nurse Ratched and her impact on the public's perception of the nursing profession is addressed. For all participants, Nurse Ratched—and her antagonistic relationship with service users—was the part of the film that stood out the most. Participants described her as a nursing archetype:

When I envision a nurse, that's what a nurse wears: the hat and the long white dresses and the white shoes and the tights (Janice)

Indeed, it was suggested that the term ‘Nurse Ratched’ has embedded itself in the public consciousness. In cases where people encounter nurses with whom they have disagreements, they would often to refer to them, pejoratively, as ‘Nurse Ratcheds’:

If there was a nurse there [my friends who don't work in mental health] didn't like, they were like, ‘Gee, she was a right Nurse Ratched’ (Nadia)

Like the name ‘Nurse Ratched’ again is a colloquial term to describe a domineering person or a person who's not a very nice person (Hector)

Participants spoke about their discomfort with the Ratched character and how she engaged with service users. Her cold, unempathetic demeanour, misuse of power and inability to build therapeutic relationships with service users was considered to be the antithesis of what participants had learned as part of their healthcare education:

I think that the nurse's communication skill was not appropriate at all. There was a non‐therapeutic relationship with patients. Now I can say that. She was very strict. Not flexible. Not understanding. She has… kind of lack of empathy throughout the movie (Melike)

Like completely the antithesis, almost of like what we are told we need to be as mental health nurses. So, kind of very unfeeling, very cold, manipulative. Not very person‐centred in any way, shape or form (Chris)

Although one participant argued that Nurse Ratched could not exist today due to safeguards in place, many others believed she still exists in some mental health services. Despite there being better governance within contemporary mental health services, there are still those who view themselves as an authority figure and will misuse their perceived position of power:

I have come across a few Nurse Ratcheds my time (Ivy)

There's kind of, like, I suppose this authoritarian kind of figure, you know, just abuse of power. Definitely a case as well of, ‘take the medication. There's a routine: stick to it’ (Oliver)

Indeed, it was suggested that all mental health staff have the potential to misuse their perceived power. As such, participants commented on the importance of self‐awareness and ensuring that they do not promote toxic hierarchies:

There's a little Nurse Ratched and all of us trying to get out, and we have to keep that little Nurse Ratched in check (Dylan)

I don't want to treat service users the way they were treated in the movie or in the old mental health unit. So I've got to catch myself before I get too stuck in my ways about this (Gina)

It was felt that this is being achieved due to the better standards of education that healthcare staff are exposed to today. In contemporary education systems, participants felt that there is a greater focus on person‐centred approaches to care:

I think it's far better education out there and far better education on how to treat patients and how to nurse (Nadia)

Some participants with nursing backgrounds expressed the substantial difference they had observed where staff have discipline‐specific mental health education compared to those with generic nurse education. It was observed that those who had undertaken discipline‐specific nursing programmes had a much better understanding of mental health and adopted a ‘less strict’ approach when engaging with service users:

Nurses [in Australia] are all registered nurses, general nurses, and then they do a 12‐month postgrad in mental health whilst working full time as a nurse. So really the quality of mental health training is terrible (Ivy)

And some even very strict, very strict to towards patients. They [those with generic nurse education] are not very expertise in the field, maybe even they didn't get any higher education in mental health. They are, they are just graduated in from nursing (Melike)

### 4.4 Theme 4. We Have Come a Long Way but We Still Have a Long Way to Go

Under this theme, the progress we have made in mental healh care is addressed, as are the improvements still required. Participants commented that current mental health care environments are more aesthetically pleasing, less clinical, more colourful and more modern than the hospital depicted in *One Flew Over the Cuckoo's Nest*. Moreover, they believed that most care has moved outside of the hospital environment and into the community:

A good few of those patients would be treated in the community as opposed to being treated in an inpatient unit. Possibly all of those patients would be in the community today (Anthony)

Relationships between staff and service users were regarded to be much more positive in a modern context, with staff expressing genuine interest in those under their care:

I feel nowadays there is more rapport between the nursing staff and the patients in a positive way (Hector)

Despite assertions that mental health care has changed for the better, there were some concerns expressed about how *One Flew Over the Cuckoo's Nest* mirrors some current practices. In this regard, participants felt that mental health care could sometimes be categorised as regressive rather than progressive:

There are still some disturbing parallels (Esme).

There was a suggestion that, despite efforts by mental health staff to help others, harm sometimes can come to service users. This was as a result of a sometimes unwaivering reverenace to the orthodoxy, an ingrained inflexibility and a tendency to default to social control:

I think the themes and some of the practices are actually an accurate representation of even some of the some of the ideas today. And if you look at that kind of psychiatry as a means of social control, the dominance of an orthodoxy, iatrogenic harm, the fact that maybe psychiatry, arguably is more harmful than good (Anthony)

Although it was acknowleged that an effort to maintain human rights is upheld, and there is relevant mental healh legislation in place, people's dignity can often be compromised in the context of power imbalances and the perpetuation of coercive and restrictive practices. Such care was considered almost ‘Victorian’ by participants:

I think very much the power lies with the clinicians. And it still does, even though we talk about it, I think that the the coercive practices are still there. I think restrictive practices are still there. […] We can still incarcerate people against the will. We can restrict visitors, phones, we can lock doors (Lydia)

I think in certain situations that it can be a little bit more, I suppose, Victorian, like we're a little bit more like the nurse kind of giving instructions as opposed to being more equal. I know that's not how it's meant to be (Nadia)

### 4.5 Theme 5. The Legacy of *One Flew Over the Cuckoo's Nest* Today

The final theme addresses the legacy and relevance of the film today. Some participants commented that the film is dated and not as accessible and relatable to a modern audience. As such, it was felt that the effect of the film is waning over time:

I told a few people that I was doing this today and they were either, like, ‘Oh yeah, I've heard of it. But I've never watched it’ or, ‘Oh, I've never even heard of that’ (Gina)

Moreover, owing to more positive media messages and promotional campaigns, several participants expressed that a contemporary audience is much more mental health‐informed than the audiences first exposed to *One Flew Over the Cuckoo's Nest* in 1975. Consequently, the film would be viewed more in the context of a historical document than a reflection of mental health care today:

I certainly think that yeah, the kind more positive messaging about accessing mental healthcare and support is certainly more to the fore now (Esme)

On the other hand, other participants commented that the film is still used as a reference point by the general public. They felt that, when the public have no experiences with mental health services, they tend to still draw on the imagery depicted in *One Flew Over the Cuckoo's Nest*:

I think it's probably the most defining piece of material that people who don't have any understanding or knowledge mental health services use as a point of reference as to what happens in contemporary mental services (Hector)

Some participants suggested that modern audiences may not consume the film in its original intended form. However, they highlighted that shorter clips of the film are shared on platforms such as *YouTube* and *TikTok*, and certain still images have been adopted through ‘meme culture’. In this regard, the messages of the film continue to reach younger audiences, albeit in a different format:

There are some memes now and people have like a screenshot of a movie and share it and don't actually know what the movie is but they understand the context of the image (Brian)

Participants gave examples of cases where service users expressed dissatisfaction with the care they were receiving or frustration with their involuntary status. In such instances, service users often drew comparisons to *One Flew Over the Cuckoo's Nest*, indicating that the film continues to cast a shadow on how mental health care is perceived:

A patient would be under a status where they wouldn't be allowed to leave the facility. And they'd be like, ‘Jesus, this is like One Flew Over the Cuckoo's Nest’ (Oliver)

He's like, ‘You're part of One Flew Over the Cuckoo's Nest. You guys are just like the movie. There's Nurse Ratched over there’ (Brian)

Even when people have not seen the film, there was a sense that it has insidiously influenced current media narratives, including the recent Netflix prequel show, *Ratched*. Indeed, several participants commented on how *Ratched* has exaggerated elements of *One Flew Over the Cuckoo's Nest*, amplifying stigma for today's audience:

This [Ratched show] really isn't doing a lot for stigma. […] And certainly, it had kind of horror kind of elements to it. And it was very dramatic and very sensational and all that sort of stuff. So, I think that one very much kind of probably portrayed psych units in a very bad light (Dylan)

Rather than conduct field research on the state of current mental health services, participants believed that people working in entertainment media instead rely on often inaccurate, outdated images they have gleaned from older texts, including *One Flew Over the Cuckoo's Nest*. As such, modern entertainment media perpetuates a myth that mental health care environments are old‐fashioned and give a sense of a custodian service:

Some of the games we play like, you know, the asylums are very much built on the kind of look of the asylum from One Flew Over the Cuckoo's Nest, you know, these long corridors, you know…. Kind of jail, almost jail looking things you know, so I definitely think it spills over… for sure… It has to have an influence. It's such an influential movie (Oliver)

One participant spoke about service users who distinguished between their initial perceptions of mental health services and the reality they experienced upon engagement. Their initial reluctance to engage with mental health services owed to stigmatising perceptions, fueled by what had been seen in movies. This participant felt that film such as *One Flew Over the Cuckoo's Nest* or other media narratives influenced by the film continue to dominate the public discourse surrounding mental health:

‘It's not at all how I thought it was going to be’, and when I asked, ‘follow that up with what made you think it was going to be in a particular way’, ‘Oh, what you'd see in movies’ (Kirsty)

Despite some of the reservations about the film and how it has impacted current perceptions of mental health services, participants commented that it should still be screened for audiences today. However, it should be clearly communicated that this is a work of fiction and does not represent contemporary services, with an emphasis on ‘look how far we have come’:

I think it can be like as a movie to retrospectively look back on and again see how far we've kind of come and how far we need to still continue to go: It's important. Sometimes it's important to look back to go forward (Nadia)

## 5 Discussion

The aim of this paper was to explore mental health staff's perspective of *One Flew Over the Cuckoo's Nest* on its 50th anniversary. According to participants in this study, the film continues to influence how the public view mental health care, either directly, but also more subtly, in that it has negatively influenced the media narratives which are consumed by contemporary audiences, including recent films and TV shows, but also social media. Such negative media exposure can influence the public's views of mental health and exacerbate stigma (Parrott 2023; Perciful and Meyer 2017; World Health Organization 2025). Participants in the current study acknowledged that more positive media messaging and promotional campaigns have led to better perceptions of mental health. While this may be true for how the public view mental illnesses/mental health challenges such as depression and anxiety (Tam et al. 2024), mental health staff and mental health care environments continue to be viewed through a sensationalised, stigmatised lens (Bone and Marchant 2016; Goodwin et al. 2023, 2024; Hajizadeh et al. 2024). Indeed, owing to negative or frightening depictions of staff and mental health care environments within media, a reluctance to seek help for mental health challenges has been reported (Goodwin et al. 2023; Srivastava et al. 2018; Wong et al. 2020). Reducing mental health stigma is important (Hajizadeh et al. 2024; Whitley and Campbell 2014). Given the success of mental health promotional campaigns on how the public view mental health challenges, similar strategies should be employed to reduce the stigma surrounding mental health staff and care environments.

Stigma in mental health is a form of social oppression stemming from the power imbalance between those who stigmatise and those who are stigmatised (Arboleda‐Flórez and Stuart 2012). Although the current landscape for human rights in mental health has improved, this dual relationship between power and stigma in mental health care exacerbates the self‐stigma felt by service users (Mertens et al. 2025). Service users with lived experiences consider mental health professionals to be key contributors to stigma, and although international agreements explicitly call for the reduction or elimination of restrictive and coercive practice in mental health care, these are considered regular healthcare acts (Savage et al. 2024).

Foucault's analysis and criticism of institutions is particularly relevant here. His concept of biopower, introduced as a response to his reflection on how ‘prisons resemble factories, schools, barracks and hospitals, which all resemble prisons’ (Foucault 1975, 228) refers to the regulation and control of individuals through surveillance and disciplinary mechanisms. This framework helps explain how modern psychiatric systems can continue to reproduce control and conformity. Although times have changed since Foucault examined these power hierarchies, current mental health systems may continue to perpetuate surveillance and dependency through coercive interventions. This internalisation of such surveillance can lead service users to monitor and correct their behaviour to align with institutional expectations, reinforcing self‐stigma (Corrigan et al. 2015; Corrigan and Rao 2012; Foucault 1975).

Power imbalances in mental health care may persist due to the resistance to change from existing institutional structures and hierarchies. These can limit service users' ability to participate meaningfully in decisions about their own care (Mertens et al. 2025). To empower service users, policy changes and cultural shifts within services in the direction of valuing lived experience may improve engagement with services (Corentin et al. 2023; Laugharne and Priebe 2006). This empowerment can be enacted at an individual level or a societal level, where the responsibility can be assumed by media productions. As discussed in this paper, media institutions play a significant role in reinforcing or challenging hierarchies. Promoting a more collaborative approach can increase awareness of the significance of language in these forms of media and practice by transferring power to people with lived experience of mental health challenges so that they can influence narratives, language and decision‐making in such representations (Hine et al. 2024; Hui and Stickley 2007).

Misuse of power also extended to the Nurse Ratched character who spoke down to service users, lacked empathy and demonstrated inadequate communication skills. Having worked with graduates who undertook both discipline‐specific mental health and more generic education, participants felt that discipline‐specific mental health education mitigated against Ratched‐like staff in practice, while more generic training resulted in less capable staff. There is evidence that a lack of mental health training for healthcare staff results in anxiousness and fear when interacting with those who experience mental health challenges, leading to social/clinical distance. It can also lead to service users receiving less effective treatment (Knaak et al. 2017). Despite this evidence, nursing programmes in several countries that previously offered discipline‐specific mental health education have reverted to a more generic approach (Lakeman et al. 2023, 2024; Warrender 2025). This has led to a model of education that ‘is not fit‐for‐purpose’ (Lakeman et al. 2024, 94) and graduates with ‘inadequate training to treat patients with mental health conditions’ (Lakeman et al. 2023, 516). Maintaining discipline‐specific mental health education is important in ensuring service users receive adequate care without prejudice and mitigating the risk of seeing ‘Nurse Ratcheds’ in practice.

This study has limitations. Although participants worked in a variety of areas, inclusion education, community and acute, most came from nursing backgrounds. We also acknowledge the potential for self‐selection bias, as participants may have had personal reasons for partaking in interviews.

## 6 Conclusion

Despite being over 50 years old, not everything from *One Flew Over the Cuckoo's Nest* has been confined to the history books. While its direct impact appears to be waning, it still has some hold over how the public view mental health care, due to its influence on contemporary media naratives. Media campaigns, similar to those used to dispel myths about mental health challenges, should be used to promote better images of mental health staff and care environments. More focussed mental health education is required for those entering a career in mental health.

## 7 Relevance for Clinical Practice

Mental health nurses need to be aware of the influence that *One Flew Over the Cuckoo's Nest* continues to have on how the public and service users view mental health care. It is vital that we take a progressive approach to care, with an emphasis on reducing power dynamics between staff and service users.

## Author Contributions

John Goodwin conceptualised the study, collected data, contributed to data analysis, contributed to drafting the article and approved the final version of the article. Katerina Drakos contributed to data analysis, contributed to drafting the article and approved the final version of the article.

## Funding

The authors have nothing to report.

## Conflicts of Interest

The authors declare no conflicts of interest.

## Supporting information


**Data S1:** inm70181‐sup‐0001‐DataS1.docx.

## Data Availability

The data that support the findings of this study are available on request from the corresponding author. The data are not publicly available due to privacy or ethical restrictions.
