# Supplementary material for: Fifty Years of One Flew Over the Cuckoo's Nest: A Qualitative Exploration of Mental Health Staff's Perspectives
Source: Int J Ment Health Nurs. 2025 Nov 25;34(6):e70181. doi: 10.1111/inm.70181 (PMC12645358; doi:10.1111/inm.70181)
Supplement: Supplementary file 1 — Data S1: inm70181‐sup‐0001‐DataS1.docx. [file INM-34-0-s001.docx]

**Interview Guide**

Can you tell me when you first saw the film “*One Flew Over the Cuckoo’s Nest*” and what struck you about it? **(prompt: before or after training/education in mental health)**

Historically, how do you think *One Flew Over the Cuckoo’s Nest* has impacted on how the general public viewed the mental health services? **(prompts: mental health care, mental health staff, service users)**

What about today - do you think that *One Flew Over the Cuckoo’s Nest* continues to have an influence on how the public view mental health and mental health services?

Do you think that *One Flew Over the Cuckoo’s Nest* has influenced current media narratives? **(i.e., are current TV/film depictions of mental health still influenced by the film?)**

When there have been other films made depicting mental health (e.g., *Joker*, *A Beautiful Mind*, *Shutter Island*) why, 50 years later, do you think *One Flew Over the Cuckoo’s Nest* continues to be such an influence or cultural reference point?

Have you seen *Ratched* on Netflix? **(Has that continued the legacy of the film?)**

If they were to remake *One Flew Over the Cuckoo’s Nest* today, a version set today, and if you were employed as an advisor, what you advise them to change so it reflects a modern mental health context? **(Prompts: community, other staff members)**

Is there value in showing the film to an audience today?

Is there anything you want to say about *One Flew Over the Cuckoo’s Nest* that we didn’t get a chance to cover?
